# Identifying factors influencing HIV testing behavior among high-risk elderly men: a mixed-methods study incorporating structural equation modeling and thematic framework

**DOI:** 10.1186/s12879-026-14305-3

**Published:** 2026-08-28

**Authors:** Yalan Wang, Renhai Tang, Qunbo Zhou, Yuecheng Yang, Song Duan, Ziyi Yuan, Qishu Wu, Junjie Wang, Duo Shan

**Affiliations:** 1 National Center for AIDS/STD Control and Prevention, Chinese Center for Disease Control and Prevention & Chinese Academy of Preventive Medicine, Beijing, China; 2 AIDS Prevention and Control Department, Dehong Dai and Jingpo Autonomous Prefecture Center for Disease Control and Prevention, Mangshi, Yunnan Province China

**Keywords:** Older men, HIV/AIDS, HIV testing, Ever-tested status, Mixed-methods study

## Abstract

**Background:**

Population ageing has been accompanied by increasing HIV risk among older adults. Timely diagnosis and treatment are essential for improving health outcomes. However, over 65% of HIV cases among older men at elevated risk in China are diagnosed at an advanced stage, largely due to delayed testing. Understanding the factors associated with prior HIV testing among this population is therefore important for promoting earlier diagnosis and developing targeted interventions.

**Methods:**

An explanatory sequential mixed-methods study was conducted in Dehong Prefecture between 2024 and 2025. The quantitative phase comprised a cross-sectional survey. Descriptive analyses were used to summarize participant characteristics, and structural equation modeling (SEM) was performed to examine the direct, indirect, and total associations between psychosocial factors and ever-tested status. In the subsequent qualitative phase, in-depth interview data were analyzed using thematic framework analysis to explain and contextualize the quantitative findings.

**Results:**

A total of 300 participants were included in the quantitative survey, and 30 participated in qualitative interviews. Among the 300 participants, the mean age was 57.2 years, and 57.7% had ever been tested for HIV. Risky sexual behaviors, particularly inconsistent condom use, were common, with reduced sexual pleasure being the main reported reason for condom non-use. In the final SEM, threat perception showed the strongest positive total association with ever-tested status (0.349; *p =* 0.001), followed by subjective norms (0.295; *p =* 0.003) and self-efficacy (0.199; *p <* 0.001). Testing belief had a positive indirect association (0.042; *p <* 0.001), whereas HIV-related information and behavioral skills showed negative total associations (-0.175; *p =* 0.009; -0.211; *p =* 0.015, respectively). Qualitative findings suggested that the negative association with HIV-related information reflected participants’ distrust of conflicting information, its limited perceived relevance, and fear-oriented messaging. They also highlighted insufficient knowledge of testing procedures, low confidence in accessing services, and age-related perceptions of HIV risk.

**Conclusion:**

Prior HIV testing among older men at elevated risk was associated with threat perception, subjective norms, self-efficacy, testing belief, the interpretation of HIV-related information, and practical testing barriers. Age-sensitive interventions should provide credible and personally relevant information, strengthen social support and self-efficacy, and offer clear, practical guidance on how and where to obtain HIV testing.

**Supplementary Information:**

The online version contains supplementary material available at https://doi.org/10.1186/s12879-026-14305-3.

## Background

The global aging population has led to an increasing number of elderly adults at risk of HIV infection [1]. Timely HIV diagnosis and treatment are essential for improving health outcomes and quality of life among older men living with HIV. However, many HIV infections among elderly men are not identified until a late stage [2]. In China, the proportion of late HIV diagnosis among elderly men is alarmingly high, with more than 65% of cases being identified at an advanced stage [3]. Delayed HIV testing may be one of the key contributors to late diagnosis among older men [4]. Compared to younger populations, elderly men are less likely to undergo voluntary HIV testing, with most diagnoses occurring through routine medical visits or hospitalization for other conditions [5]. Despite government efforts to expand HIV testing coverage, elderly men continue to face persistent social and structural obstacles that hinder voluntary testing [6], including social misconceptions that HIV is a “young person’s disease,” discomfort among healthcare providers when discussing sexual health with older patients, and fear of discrimination [7, 8]. Previous studies have predominantly focused on younger key populations, such as men who have sex with men (MSM) and female sex workers [9–11], leaving elderly high-risk men underrepresented in research and intervention strategies. Evidence regarding the psychological, behavioral, and social factors associated with HIV testing in this population therefore remains limited [2].

Theory-based behavioral interventions can effectively promote health behavior change, and behavioral theories grounded in psychological and social perspectives provide important frameworks for understanding health-related behaviors [12, 13]. Accordingly, the Information-Motivation-Behavioral Skills (IMB) framework has been increasingly applied to studies of HIV testing behavior [14, 15]. The model proposes that HIV-related health behavior is shaped by relevant information, personal and social motivation, and the behavioral skills required to perform the behavior [16–19]. However, HIV testing among older men may also be influenced by age-related misconceptions, limited familiarity with testing services, concerns about stigma, and reliance on family members or healthcare providers. The broad components of the IMB model may therefore benefit from further specification to reflect the motivational and capability-related processes involved in HIV testing among this population. To support this specification, concepts from self-determination theory (SDT) concerning autonomy, competence, and relatedness were considered during initial item development and conceptual refinement, thereby broadening the coverage of relevant motivational and capability-related experiences [20, 21]. In the extended IMB framework used in this study, personal motivation was represented by testing belief and threat perception, social motivation by subjective norms, and behavioral skills by testing self-efficacy and perceived testing-related barriers. Thus, compared with the original IMB model, the present study extended the framework by disaggregating the broad motivation component into theoretically distinct personal and social motivational factors and by further differentiating behavioral skills into practical skills and self-efficacy, thereby providing a more comprehensive framework to examine how these interconnected factors were jointly associated with HIV testing among high-risk elderly men.

Additionally, the integration of quantitative and qualitative evidence can provide a more comprehensive understanding of health behaviors [22, 23]. Quantitative analysis can identify the principal associations and potential pathways between psychosocial factors and HIV testing, whereas qualitative inquiry can clarify the experiences and contextual circumstances underlying these associations. Guided by the extended IMB framework, this mixed-methods study therefore examined psychosocial factors associated with having ever undergone HIV testing—operationalized as ever-tested status—among older men at high risk of HIV infection in China and explored the contextual explanations for the observed associations. The findings may inform the development of more accessible, acceptable, and age-appropriate HIV testing strategies and contribute to reducing late HIV diagnosis in this population.

## Methods

### Study design

This study adopted an explanatory sequential mixed-methods design. The quantitative phase was conducted first to identify key factors and pathways associated with ever-tested status among high-risk elderly men using SEM. For the qualitative phase, participants were purposively selected from the quantitative sample. Based on the SEM results, participants were classified into subgroups by combining ever-tested status with the key variables involved in statistically significant or unexpected SEM associations. This approach allowed the study to include participants from different model-informed subgroups, including those whose ever-tested status was consistent with the expected pattern and those whose status deviated from it. Interview questions were guided by a common semi-structured framework, but specific probes were tailored to participants’ subgroup characteristics. This approach enabled further exploration of the observed quantitative patterns and the individual, psychological, and contextual factors associated with ever-tested status.

### Participants

Participants were recruited from Dehong Prefecture, Yunnan Province, China, which has successfully achieved the “90-90-90” HIV control target. Eligible participants were men aged 50 years or older who engaged in commercial sex or other high-risk behaviors in the past 12 months. Inclusion criteria were: (1) aged ≥ 50 years; (2) reported at least one HIV-related high-risk sexual behaviour in the past 12 months, including commercial sex, multiple sexual partners, sex with casual or non-regular partners, or condomless/inconsistent condom use during sexual intercourse; (3) reported either being HIV-negative or never having undergone HIV testing; (4) willingness to participate and provide informed consent. Exclusion criteria included cognitive impairment, severe mental disorders, or active substance dependence.

Participants received a gift valued at 10 Chinese Yuan (CNY; approximately US$1.47) after completing the structured questionnaire. Those who participated in the in-depth interviews received an additional 200 Chinese Yuan (approximately US$29.24) in compensation for their time.

## Data collection

### Quantitative data collection

From September to December 2024, 300 participants were recruited using purposive convenience sampling through local community-based organizations (CBOs) and sexual health clinics. Potential participants were identified during routine outreach or clinical service provision and screened according to the eligibility criteria. Eligible individuals were invited to participate, and those who agreed provided written informed consent before completing the survey. Participants were informed that their responses would be kept confidential and used only for research purposes. Interviews were conducted in a private setting to protect participants’ privacy and encourage honest reporting of sensitive information. Trained interviewers conducted face-to-face structured surveys, covering:

1. **Sociodemographic Characteristics** Sociodemographic variables included age, ethnicity, marital status, education, occupation, monthly income, household registration (urban or rural), local residence. Ever-tested status was assessed by asking participants whether they had ever undergone HIV testing. Responses were coded as 1 for ever tested and 0 for never tested, regardless of whether the test was voluntary, provider-initiated, or conducted through another service setting.
2. **HIV-Related Information** In this study, HIV-related information was operationalized through an 8-item questionnaire adapted from the National HIV Sentinel Surveillance Implementation Manual [24]. Each item had three response options: “correct,” “incorrect,” or “don’t know.” A correct response was assigned 1 point, and incorrect or “don’t know” responses were assigned 0, yielding a total score ranging from 0 to 8. A total score of ≥ 6 was defined as adequate knowledge. In the SEM analysis, HIV-related information was entered as a binary variable indicating adequate versus inadequate knowledge.
3. **Sexual Health Status and High-risk Sexual Behaviors** Sexual health status and high-risk sexual behaviours were assessed using self-reported questions. Sexual health status included self-perceived usual sexual desire, frequency of sexual activity, erectile function, duration of intercourse, use of sexual enhancement medications, andrological diseases, and satisfaction with sexual needs. Sexual risk behaviours were assessed separately by partner type, including fixed female partners, non-regular female partners, and commercial female partners. For each partner type, participants reported sexual activity, number of partners, frequency of intercourse, condom use during the most recent sexual intercourse, and reasons for condom non-use. Questions concerning commercial sex additionally assessed the venues where participants contacted commercial partners and the average amount paid per encounter.
4. **Development and refinement of the extended IMB measurement model** A 30-item scale was developed by integrating the IMB and SDT frameworks. Items were generated through literature review and theoretical mapping, followed by two rounds of Delphi consultation with experts to ensure content validity. A pilot study was subsequently conducted to assess item clarity and preliminary psychometric performance, and minor revisions were made accordingly. All items were rated on a 5-point Likert scale ranging from 1 (completely disagree) to 5 (completely agree). The original scale was designed to broadly assess SDT- and IMB-informed factors potentially associated with ever-tested status. Before specifying the final structural model, theoretically relevant items were mapped to the constructs examined in the extended IMB framework, and the proposed measurement model was subsequently evaluated using confirmatory factor analysis (CFA). Items with insufficient theoretical correspondence or standardized factor loadings below 0.50 were excluded [25, 26]. Construct reliability and convergent validity were subsequently evaluated using composite reliability (CR) and average variance extracted (AVE) respectively, with values of CR ≥ 0.70 and AVE ≥ 0.50 considered acceptable. Finally, 14 items were retained as indicators of the latent constructs in the SEM. All retained items had standardized factor loadings greater than 0.50. Furthermore, the CR and AVE values for each construct exceeded the recommended thresholds, supporting the reliability and convergent validity of the measurement model (Supplementary Table 1):

#### **Subjective norms**

represented the social motivation component and reflected participants’ perceived availability of community-based support and peer-support referral information related to HIV testing and post-test follow-up services. This construct was measured using two items and its Cronbach’s α was 0.907.

#### Threat perception

reflected participants’ perceived threat of HIV infection and the potential consequences of delayed testing. It was measured using three items assessing perceptions of the severity of the local HIV epidemic, the risk of delayed HIV diagnosis and treatment if testing was not performed in time, and the possibility of unknowingly transmitting HIV to others without timely testing. The Cronbach’s α for this construct was 0.847.

#### Belief

reflected participants’ positive beliefs about the value and responsibility of HIV testing, including its role in confirming one’s health status and fulfilling responsibility toward oneself and others. This construct was measured using three items and demonstrated good internal consistency, with a Cronbach’s α of 0.899.

#### Behavioral skills

focused on actual difficulties or behavioral obstacles that may hinder testing behavior, with higher scores indicating lower perceived skill or capability [27]. Scores were interpreted in the reverse direction, with higher scores indicating greater testing-related difficulties and lower perceived behavioral skills or capability. The construct was measured using three items and demonstrated an acceptable Cronbach’s α of 0.760.

#### Self-efficacy

reflected participants’ perceived confidence in accessing HIV testing information, completing HIV self-testing procedures, and understanding key issues related to test interpretation. This construct was measured using three items and demonstrated good internal consistency, with a Cronbach’s α of 0.922.

### Qualitative data collection

From January to February 2025, 30 participants were purposively selected from the quantitative sample based on the quantitative findings. Equal representation of ever-tested and never-tested participants was ensured, with 15 participants in each group. Within each group, participants were further selected to capture variation in key characteristics involved in significant or unexpected SEM associations, such as information, self-efficacy, and behavioral skills. Participants whose characteristics and ever-tested status were either consistent with or different from the overall SEM patterns were included to facilitate further exploration of the quantitative findings. The interviews explored three broad areas: (1) cognitive and emotional barriers to HIV testing; (2) social and cultural factors related to testing decisions; and (3) perceived benefits and risks of HIV testing. Although all interviews followed this common framework, additional questions were tailored to participants’ ever-tested status and psychosocial characteristics.

## Data analysis

### Quantitative data analysis

Descriptive analyses were first conducted to summarize the characteristics of the study participants. Differences in demographic characteristics between urban and rural participants were examined using independent-samples t tests for continuous variables and chi-square tests for categorical variables. Frequencies and percentages were used to describe sexual activity, sexual health characteristics, and high-risk sexual behaviors. All tests were two-sided, with *P* < 0.05 considered statistically significant.

The initial SEM was specified based on the extended IMB framework and estimated using AMOS version 24.0 with maximum likelihood estimation. Ever-tested status was coded as 0 = no and 1 = yes and entered into the SEM as an observed endogenous variable. It was treated as an observed numerical variable without applying a logit or probit link function because the analysis focused on estimating and comparing the standardized direct, indirect, and total associations between the psychosocial constructs and ever-tested status within the same covariance-structure model. This analytical approach was also informed by previous AMOS-based SEM studies that modeled comparable binary outcomes as 0/1 observed variables [28, 29]. To obtain a more parsimonious model, nonsignificant structural paths were removed sequentially only when their removal was theoretically justifiable and did not compromise the theoretical coherence or overall interpretation of the model. Model fit was evaluated using the ratio of chi-square to the degree of freedom (χ²/df), the comparative fit index (CFI), the Tucker–Lewis index (TLI), and the root mean square error of approximation (RMSEA) with its 90% confidence interval. These indices were considered collectively when evaluating overall model fit. Statistical inference for the direct, indirect, and total effects was based on bootstrap standard errors and bias-corrected 95% confidence intervals obtained from 5,000 resamples.

To assess the potential influence of sociodemographic characteristics, a covariate-adjusted sensitivity analysis was conducted by re-estimating the SEM with age, urban/rural household registration, education, occupation, monthly income, and local residence included as direct predictors of ever-tested status. The original measurement structure and principal structural pathways were retained to facilitate comparison with the main analysis.

### Qualitative data analysis

Qualitative data were analyzed using thematic framework analysis, with the initial analytical framework informed by the quantitative findings and the interview guide developed from participants’ model-derived profiles. All interviews were audio-recorded and transcribed verbatim, and the transcripts were imported into NVivo 12 for data management and coding. Two researchers independently coded the transcripts, compared coding results, and resolved discrepancies through discussion to enhance the credibility and consistency of the analysis.

## Results

### Quantitative results

#### Sample characteristics

Table 1 shows the characteristics of participants by urban-rural household. The mean age of the 300 participants was 57.18 ± 6.21 years, and urban participants were older than rural participants (58.75 vs. 56.28 years). No significant differences were found in ethnicity or marital status. Significant urban-rural differences were observed in education, occupation, monthly income, local residence, and ever-tested status. Compared with urban participants, rural participants were less likely to have a senior high school education or above (6.28% vs. 31.19%), more likely to be farmers (69.11% vs. 14.68%), less likely to have a monthly income above CNY 3,000 (18.85% vs. 59.63%), and less likely to report local residence (70.16% vs. 89.91%). The proportion of participants with ever-tested status was 57.67%, with a significantly higher rate among urban participants than among rural participants (76.15% vs. 47.12%, *p* < 0.001).

**Table 1:** Characteristics of participants in the quantitative survey by urban–rural household registration status

Variables	Total (*N* = 300)	Urban (*n* = 109)	Rural (*n* = 191)	Statistic	*P*-value
**Age**,** Mean ± SD**	57.18 ± 6.21	58.75 ± 7.07	56.28 ± 5.48	t = 3.15	**0.002**
**Ethnicity**				χ²=1.57	0.210
Han	227 (75.67%)	78 (71.56%)	149 (78.01%)		
Ethnic minorities	73 (24.33%)	31 (28.44%)	42 (21.99%)		
**Marital status**				χ²=1.41	0.235
Married/cohabiting	222 (74.00%)	85 (77.98%)	137 (71.73%)		
Unmarried/widowed/divorced	78 (26.00%)	24 (22.02%)	54 (28.27%)		
**Education**				χ²=34.07	**< 0.001**
Primary school or below	147 (49.00%)	47 (43.12%)	100 (52.36%)		
Junior high school	107 (35.67%)	28 (25.69%)	79 (41.36%)		
Senior high school or above	46 (15.33%)	34 (31.19%)	12 (6.28%)		
**Occupation**				χ²=82.26	**< 0.001**
Farmer	148 (49.33%)	16 (14.68%)	132 (69.11%)		
Others	152 (50.67%)	93 (85.32%)	59 (30.89%)		
**Average monthly income (CNY)**				χ²=52.32	**< 0.001**
≤ 1,000	61 (20.33%)	11 (10.09%)	50 (26.18%)		
1,001–3,000	138 (46.00%)	33 (30.28%)	105 (54.97%)		
> 3,000	101 (33.67%)	65 (59.63%)	36 (18.85%)		
**Local residence**				χ²=15.44	**< 0.001**
No	68 (22.67%)	11 (10.09%)	57 (29.84%)		
Yes	232 (77.33%)	98 (89.91%)	134 (70.16%)		
**Ever-tested status**				χ²=23.95	**< 0.001**
Yes	173 (57.67%)	83 (76.15%)	90 (47.12%)		
No	127 (42.33%)	26 (23.85%)	101 (52.88%)		

### Sexual activity and sexual health characteristics of participants

Among the 300 participants, 82.0% reported normal sexual desire, while 56.9% reported engaging in sexual activity two to three times per month. A total of 84.0% were able to consistently achieve and maintain an erection during intercourse, and 79.6% reported an intercourse duration of 3–10 min. Overall, 78.3% were basically satisfied with their sexual life, while 21.6% reported being basically or seriously dissatisfied. In addition, 24.2% reported andrological diseases, mainly prostate disease, erectile dysfunction, premature ejaculation, or sexually transmitted diseases. Among participants who reported unmet sexual needs (*n* = 65), the most common reasons were lack of sexual partner (41.5%), lack of sexual interest from spouse (30.8%), and insufficient sexual ability (20.0%) (Supplementary Table 2).

### High-risk behaviors

Among the participants, 231 (77.0%) reported having had sexual intercourse with a spouse or regular partner in the previous six months. Among them, 63.2% (146/231) did not use a condom during their most recent sexual encounter. The main reasons were perceiving it as unnecessary (58.9%, 86/146) and reduced sexual pleasure (30.1%, 44/146). Additionally, 56.0% (168 individuals) had sex with non-regular partners (excluding commercial partners), with 24.4% (41/168) reporting no condom use during the last encounter, primarily due to reduced sexual pleasure (56.1%, 23/41). Furthermore, among the 85.3% (256 individuals) who had engaged in commercial sex, 27 participants did not use a condom during their most recent encounter, again mainly citing reduced pleasure (66.7%, 18/27) as the reason.

### Factors associated with ever-tested status using SEM analysis

**Fig. 1:**
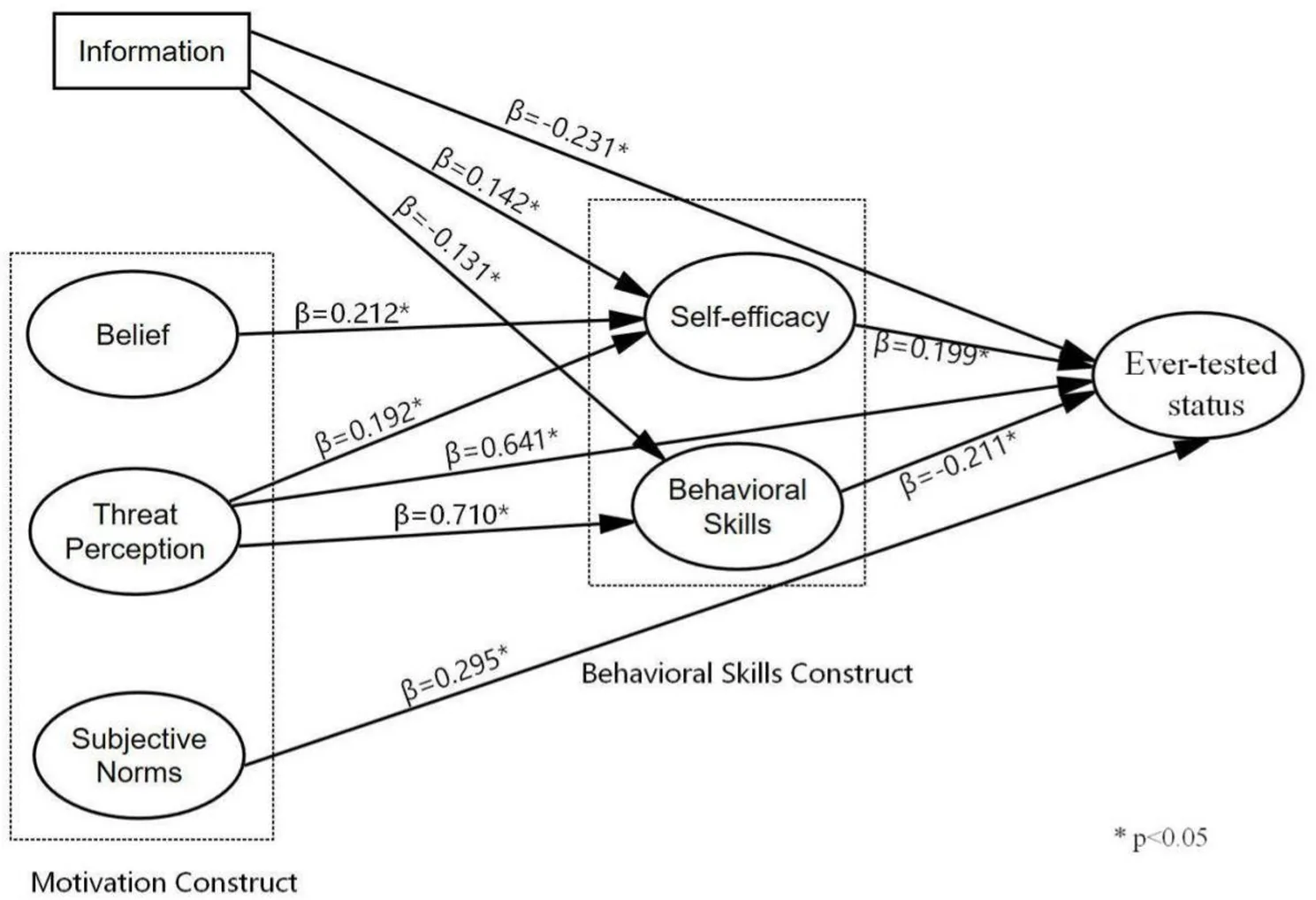
SEM of the relationships between latent constructs and ever-tested status

After fitting the initial SEM, non-significant paths were removed when theoretically justifiable, resulting in a more parsimonious final model. The final model demonstrated an acceptable fit: χ²/df = 2.630, CFI = 0.954, TLI = 0.938, and RMSEA = 0.074 (90% CI: 0.062–0.086). These indices indicated that the revised model adequately fitted the observed data and could be used to examine the direct and indirect effects of latent constructs on ever-tested status (Fig. 1).

Table 2 presents the standardized direct, indirect, and total effects among the variables included in the SEM. Threat perception had the largest positive total effect on ever-tested status (β = 0.349; *p* = 0.001), followed by subjective norms (β = 0.295; *p* = 0.003) and self-efficacy (β = 0.199; *p* < 0.001). Belief had only a small but significant indirect effect on ever-tested status (β = 0.042; *p* < 0.001). Information had a negative direct effect (β = -0.231; *p* = 0.004) and a positive indirect effect (β = 0.056; *p* < 0.001), resulting in a negative total effect (β = -0.175; *p* = 0.009). The behavioral skills score, for which lower values indicated higher levels of behavioral skills, had negative direct and total effects on ever-tested status (both β = −0.211; *p* = 0.015). The covariate-adjusted sensitivity analysis yielded findings consistent with those of the main analysis. All principal psychosocial pathways retained their original directions and statistical significance, and the small changes in the standardized coefficients did not alter the substantive conclusions. Among the sociodemographic covariates, only local residence (β = 0.123, 95% CI: 0.014–0.243; *p* = 0.025) and age (β = 0.094, 95% CI: 0.001–0.202; *p* = 0.048) were positively associated with ever-tested status. The adjusted model showed an acceptable fit (χ²/df = 2.508, CFI = 0.918, TLI = 0.891, and RMSEA = 0.071; 90% CI: 0.063–0.079) (Supplementary Table 3).

**Table 2:** Standardized direct, indirect, and total effects in the final SEM

Outcome variable	Predictor	Direct effect	*P*-value	95% CI	Indirect effect	*P*-value	95% CI	Total effect	*P*-value	95% CI
Self-efficacy	**Belief**	0.212	0.001	**[0.094**,** 0.332]**	—	—		0.212	0.001	**[0.094**,** 0.332]**
	**Threat perception**	0.192	0.010	**[0.051**,** 0.326]**	—	—		0.192	0.010	**[0.051**,** 0.326]**
	**Information**	0.142	0.001	**[0.061**,** 0.232]**	—	—		0.142	0.001	**[0.061**,** 0.232]**
Behavioral skills	**Threat perception**	0.710	**<** 0.001	**[0.602**,** 0.807]**	—	—		0.710	**<** 0.001	**[0.602**,** 0.807]**
	**Information**	-0.131	0.001	**[− 0.223**,** − 0.049]**	—	—		-0.131	0.001	**[− 0.223**,** − 0.049]**
Ever-tested status	**Belief**	—	—		0.042	**<** 0.001	**[0.016**,** 0.081]**	0.042	**<** 0.001	**[0.016**,** 0.081]**
	**Threat perception**	0.461	0.001	**[0.202**,** 0.730]**	-0.112	0.094	**[-0.336**,** 0.018]**	0.349	0.001	**[0.148**,** 0.535]**
	**Subjective norms**	0.295	0.003	**[0.109**,** 0.496]**	—	—		0.295	0.003	**[0.109**,** 0.496]**
	**Information**	-0.231	0.004	**[− 0.340**,** − 0.100]**	0.056	**<** 0.001	**[0.025**,** 0.112]**	-0.175	0.009	**[− 0.275**,** − 0.049]**
	**Self-efficacy**	0.199	**<** 0.001	**[0.098**,** 0.304]**	—	—		0.199	**<** 0.001	**[0.098**,** 0.304]**
	**Behavioral skills**	-0.211	0.015	**[-0.455**,** -0.039]**	—	—		-0.211	0.015	**[− 0.455**,** − 0.039]**

Note: Bias-corrected 95% confidence intervals for the direct, indirect, and total effects were obtained using 5,000 bootstrap resamples

Items measuring behavioral skills were reverse-scored, such that lower scores indicated higher levels of perceived behavioral skills or capability

### Qualitative results

#### Information-related barriers: distrust, low relevance, and fear-oriented messages

**Fig. 2:**
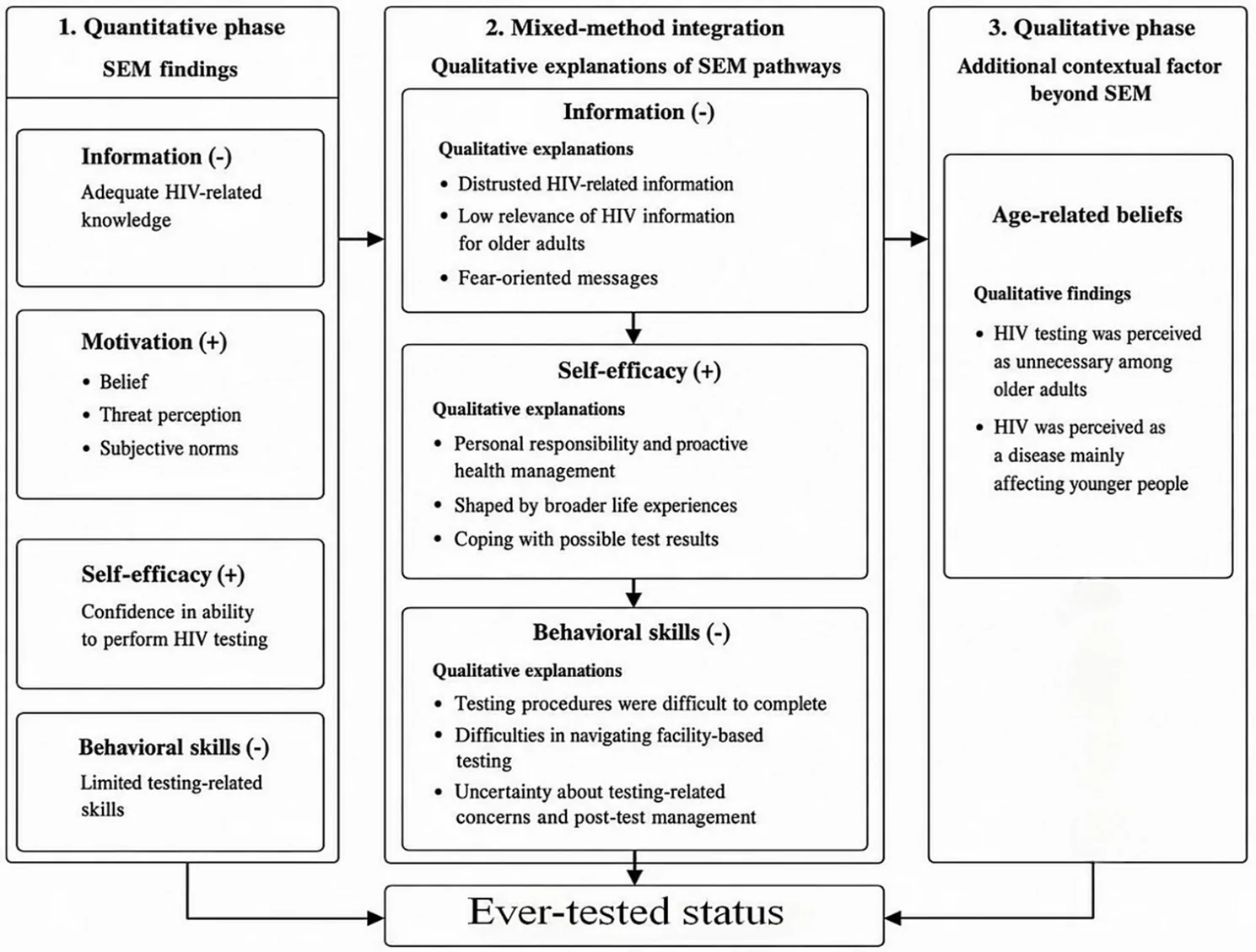
Integration of quantitative and qualitative findings explaining ever-tested status among high-risk elderly men. (+): positive associations (-): negative associations

The quantitative results showed that HIV information was negatively associated with ever-tested status, contrary to the expected positive relationship between information and HIV testing. The qualitative findings provided context for this pattern by illustrating how participants encountered and interpreted HIV-related information. Their accounts highlighted concerns about whether the information was trustworthy, understandable, relevant, and actionable (Fig. 2).

First, some participants questioned the accuracy and reliability of HIV-related information. Conflicting information from different sources made it difficult for them to judge what was credible, which in turn weakened their willingness to seek testing.

The HIV information on those flyers—I can’t tell if it’s true or not. It just makes me uneasy. (Participant 1)

When I search online, the information is all over the place. I don’t know what to believe, so I lose interest in testing. (Participant 2)

Second, several participants felt that HIV-related messages were difficult to understand or not tailored to older adults. Technical language and youth-oriented content made the information feel distant from their own lives, reducing its perceived relevance.

The pamphlets from the community are full of technical terms I don’t understand, so I lose interest in learning about testing. (Participant 3)

All the messages seem to be about young people. There’s nothing about older adults, so it doesn’t feel relevant to me. (Participant 4)

Third, participants reported that some messages overemphasized the severity and dangers of HIV while providing limited explanation of the benefits and procedures of testing. Such fear-oriented information appeared to increase anxiety rather than encourage action.

The messages only talk about how terrible it is to get infected. It scared me so much I didn’t want to get tested. (Participant 5)

I haven’t really heard about any benefits of testing. It just feels like it doesn’t matter whether I get tested or not. (Participant 6)

### Self-efficacy as a bridge from awareness to testing action

The SEM indicated that high self-efficacy was positively associated with ever-tested status among elderly at high risk. The qualitative findings provided further context for this association by illustrating how participants perceived their ability to undergo testing and cope with possible results. Participants with higher self-efficacy tended to view HIV testing as a manageable health behavior and demonstrated greater confidence in facing both the testing process and potential results.

Some participants linked HIV testing with personal responsibility and proactive health management. For them, testing was not perceived as something shameful or frightening, but as a rational step to protect their health.

I’ve always taken my health seriously. I think regular testing is being responsible, so I’m not afraid of getting tested. (Participant 7)

Other participants who were familiar with the testing process expressed less uncertainty and fear, along with greater confidence in completing the procedure and coping with possible results.

I looked into the testing process beforehand. It’s just a blood draw or a simple test—not scary. Even if the result is bad, at least I’ll know and can make a plan.(Participant 8)

In contrast, participants with lower self-efficacy often felt unable to cope with the testing process or its possible consequences. Their reluctance was not only related to fear of a positive result, but also to uncertainty about subsequent actions and limited confidence in managing social pressure or stigma after testing.

I feel like I’m too old to face the results. If something is wrong, I don’t even know what to do. (Participant 9)

People in my neighborhood say if you get tested and they find something, it’s all over. So I got scared and didn’t go. (Participant 10)

Participants’ narratives also linked self-efficacy to broader life experiences. Those who referred to their educational background or previous experience of managing illness described greater confidence in understanding health information, navigating services, and coping with possible adverse outcomes.

I’ve had some education, so I understand the importance of health and trust that I can handle HIV testing. (Participant 11, high school education)

I had a serious illness before and recovered well after active treatment. That made me feel confident I could handle HIV testing too. (Participant 12, previously treated for a chronic illness)

Overall, the qualitative findings supported and extended the SEM results by providing context for the positive association between self-efficacy and ever-tested status. Participants described self-efficacy as encompassing confidence in understanding the testing process, completing the procedure, coping with possible results, and managing related social and emotional concerns.

### Testing-related practical difficulties and concerns

Although behavioral skills showed an unexpected negative association with ever-tested status in the SEM, the qualitative findings did not indicate that stronger practical skills were associated with less testing. Instead, participants described several practical difficulties in completing HIV testing. Some participants reported that self-testing procedures were difficult to follow, particularly because of multiple operational steps, unclear instructions, and small print in the instruction manuals. Others described difficulties in navigating facility-based testing, including not knowing where to go first or how to complete the testing process at service sites.

The self-testing kit requires sampling and adding reagents—too many steps. I couldn’t figure it out, so I gave up. (Participant 13)

At the testing site, I had no idea what to do first. The staff didn’t explain clearly. I had to run around a lot, so I just didn’t want to go again. (Participant 14)

The instruction manual in the test kit is printed in such tiny font. Even with my reading glasses, I could barely read it, so I didn’t dare try it. (Participant 15)

Participants also described uncertainty about how to handle testing-related concerns, including privacy protection, communication with service providers, and coping with possible test results. These concerns made the testing process feel difficult to manage for some participants.

I was worried about my privacy during testing but didn’t know how to talk to the staff. I felt stuck, so I ended up not getting tested. (Participant 16)

If the result turned out bad, I wouldn’t know how to tell my family or what to do next. That’s why I’ve been putting it off. (Participant 17)

The qualitative findings regarding behavioral skills were not fully consistent with the SEM results. Participants who described hesitation or avoidance of HIV testing also reported practical difficulties and related concerns, including difficulty using self-testing kits, limited understanding of testing procedures, privacy concerns, and uncertainty about how to respond to possible results.

### Age-related cultural beliefs as contextual barriers to HIV testing

Although age-related beliefs were not directly represented in the SEM, the qualitative findings extended the quantitative results by identifying contextual factors relevant to HIV testing decisions. Participants’ accounts linked age-related beliefs with their perceptions of HIV risk and the need for testing. Some participants, including those living in rural areas, viewed HIV as a disease that mainly affected younger people and regarded testing as unnecessary for older adults. These views were accompanied by a low perceived need for testing despite potential exposure to HIV.

At our age, the days of being sexually active are over. What’s the point of getting tested now? (Participant 18)

AIDS is something only young people worry about. We’re old and live quiet lives—it doesn’t concern us. (Participant 19)

## Discussion

Using a mixed-methods design, this study examined psychosocial and contextual factors associated with ever-tested status among high-risk elderly men in China. The SEM showed that subjective norms, threat perception, and self-efficacy were directly and positively associated with ever-tested status, while belief had a positive indirect association. In contrast, behavioral skills and HIV-related information showed significant negative associations with ever-tested status. The qualitative findings further contextualized selected SEM associations by illustrating how participants understood HIV-related information, described their confidence in completing testing and coping with possible results, experienced procedural difficulties and privacy concerns related to testing, and expressed age-related sociocultural beliefs related to their perceived need for testing. These findings provide deeper insight into several key factors underlying HIV testing decisions among high-risk elderly men and suggest that current HIV prevention messages and service delivery approaches may not fully address the needs, experiences, and communication preferences of elderly men, particularly in an increasingly digitalized context [30]. Therefore, age-tailored interventions are needed to improve the relevance, accessibility, and acceptability of HIV testing promotion in this population.

Unlike previous studies that have generally reported positive associations between HIV-related information and HIV testing [31, 32], our SEM showed negative direct and total associations between information and ever-tested status among high-risk elderly men. The qualitative findings provided context for this unexpected pattern by identifying several information-related barriers, including distrust of information sources, difficulty understanding technical language, limited relevance to older adults, and fear-oriented messages that provided little practical guidance about testing. These findings suggest that exposure to HIV-related information alone may not be sufficient to support testing among older men. However, the observed negative associations should not be interpreted as evidence that HIV-related information reduces testing. Rather, they may reflect that the information encountered by participants did not adequately address their informational and practical needs. Therefore, HIV testing promotion in this population should move beyond general knowledge dissemination and develop age-tailored communication strategies [33]. Messages should use plain language, avoid excessive fear appeals, clearly explain the benefits and procedures of testing, emphasize privacy protection, and provide concrete post-test support pathways.

Self-efficacy and behavioral skills showed opposite associations with ever-tested status, although both were conceptualized within the broader behavioral skills domain. This finding was not fully aligned with the expected IMB pathway, in which behavioral skills are generally assumed to facilitate health behavior [15, 34]. Self-efficacy was positively associated with ever-tested status, consistent with previous studies [35]. The qualitative findings helped clarify how this association may arise among high-risk elderly men. Participants with greater self-efficacy viewed testing as manageable and felt able to cope with its results, whereas those with lower self-efficacy expressed uncertainty about the procedure and its medical and social consequences. These findings suggest that HIV testing self-efficacy extends beyond confidence in completing the test itself to confidence in managing its possible medical, emotional, and social consequences. Our findings extend previous evidence by illustrating what self-efficacy specifically involves among high-risk older men [36, 37]. The qualitative accounts also identified factors that were not included in the quantitative model, such as familiarity with testing procedures, previous experience of managing illness, anticipated stigma and social pressure, and uncertainty about post-test support. These factors should therefore be considered when interpreting the observed SEM association. Age-tailored interventions may benefit from explaining each step of the testing process, clarifying the support available after different test results, providing confidential counselling, and ensuring clear referral and treatment pathways.

In contrast, the negative association between behavioral skills and ever-tested status was unexpected [38]. The quantitative and qualitative findings regarding behavioral skills were not fully aligned. This finding may reflect a mismatch between procedural awareness and active testing behavior. In Dehong, many older adults may undergo HIV testing through healthcare contact or service-driven screening rather than through their own active test-seeking skills [39]. In this context, testing experience does not necessarily imply strong behavioral skills, while knowing where or how to test does not necessarily translate into active testing. After accounting for self-efficacy, threat perception, testing belief, and subjective norms, the remaining component of behavioral skills may mainly reflect procedural awareness rather than practical readiness for testing. Moreover, the behavioral skills scale mainly assessed whether participants knew how to access HIV testing, but did not capture their ability to understand and complete specific testing procedures. Therefore, the measured behavioral skills may have reflected general awareness or perceived ability rather than the practical skills required to complete HIV testing. This gap suggests that knowing where to access HIV testing is not enough for high-risk elderly men in Dehong. Interventions should focus on translating testing awareness into practical readiness by providing age-friendly procedural guidance, simplified service pathways, and support for post-test care.

Threat perception, subjective norms, and testing belief were all positively associated, either directly or indirectly, with ever-tested status, consistent with the motivational assumptions of the IMB framework [34]. By further distinguishing motivation into these three components, this study provided a more nuanced understanding of how motivation operates. Specifically, perceived HIV-related threat and social normative support appeared to be more directly associated with ever-tested status, whereas positive belief about testing operated through indirect pathways. Notably, threat perception showed the largest positive total association, suggesting that recognizing the necessity and urgency of HIV testing may be particularly important in this population. At the same time, encouragement from peers, family members, or community workers may reduce hesitation, normalize HIV testing, and increase confidence in seeking services. Therefore, interventions should incorporate context-appropriate social support strategies to create a more acceptable and supportive environment for HIV testing among high-risk elderly men.

### Limitations

This study has several limitations. First, the cross-sectional design precludes causal inference, while self-reported sexual behaviors and ever-tested status may be affected by recall and social desirability biases. Second, non-probability recruitment in Dehong, with its distinct HIV epidemic and long-standing testing programs, may have introduced selection bias and limits the generalizability of the findings to older men in other settings. Third, although the newly developed scale showed acceptable reliability and validity, it requires validation in larger and more diverse populations. In particular, the behavioral skills dimension primarily assessed participants’ perceived capability and practical challenges related to HIV testing, while some specific operational aspects of completing the testing process may not have been fully captured. Future studies could further refine this dimension by incorporating additional items related to practical testing experiences among older adults. Fourth, despite the stability of the main SEM pathways in the covariate-adjusted sensitivity analysis, unmeasured sociodemographic and contextual factors may still have influenced the observed associations. Future studies should further validate the scale and incorporate a broader range of relevant factors. Fifth, treating the binary ever-tested status as a numerical variable without applying a categorical link function may have reduced the precision of the estimated associations. However, this study primarily aimed to identify psychosocial factors associated with ever-tested status and their potential pathways rather than to precisely estimate individual testing probabilities. Despite this limitation, the findings provide useful evidence for understanding HIV testing among older men at high risk of HIV infection and for informing the development of targeted interventions.

## Conclusions

By integrating quantitative and qualitative findings, this study identified psychosocial and contextual factors associated with ever-tested status among high-risk older men and provided evidence to inform age-appropriate HIV testing interventions. Such interventions may benefit from delivering understandable and relevant information, fostering supportive social norms, and strengthening confidence in seeking and completing testing. Framing HIV testing as part of responsible health management may further enhance its relevance to older men. Practical guidance should clearly explain where and how to obtain testing, how privacy is protected, and what follow-up support is available. Overall, improving HIV testing uptake among high-risk elderly men requires an integrated, age-sensitive approach that addresses cognitive, motivational, psychological, and practical barriers throughout the testing process.

## Supplementary Information

Below is the link to the electronic supplementary material.

Supplementary Material 1

Supplementary Material 2

Supplementary Material 3

## Data Availability

All data essential to the conclusion are included in this manuscript and additional data are available upon reasonable request from the first author.

## Abbreviations

SEM: Structural Equation Modeling
MSM: men who have sex with men
IMB: Information-Motivation-Behavioral Skills
SDT: Self-Determination Theory
CNY: Chinese Yuan
NCAIDS: National Center for AIDS/STD Control and Prevention, Chinese Center for Disease Control and Prevention
CDC: Chinese Center for Disease Control and Prevention
USD: United States Dollar
CBOs: community-based organization
CI: Confidence interval
CFA: Confirmatory factor analysis
CR: Composite reliability
AVE: Average variance extracted
RMSEA: Root mean square error of approximation
CFI: Comparative fit index
TLI: Tucker–Lewis index
SD: Standard deviation
ML: Maximum likelihood
