# Supplementary material for: Identifying factors influencing HIV testing behavior among high-risk elderly men: a mixed-methods study incorporating structural equation modeling and thematic framework
Source: BMC Infect Dis. 2026 Aug 28;26:1671. doi: 10.1186/s12879-026-14305-3 (PMC13587304; doi:10.1186/s12879-026-14305-3)
Supplement: Supplementary file 1 [file 12879_2026_14305_MOESM1_ESM.docx]

Supplementary Table 1 Items retention and exclusion decisions and psychometric properties of the measurement model

| **SDT Dimension** | **Item code** | **Proposed construct in the initial theoretical mapping** | **Scale item** | **Status** | **Reason for exclusion** | **Standardized factor loading** | **CR** | **AVE** |
| --- | --- | --- | --- | --- | --- | --- | --- | --- |
| **Autonomy** | A1 | **Motivation-Personal belief** | HIV testing facilitates early diagnosis, and early diagnosis allows for effective treatment. | Excluded | Standardized factor loading below 0.50 |  |  |  |
|  | A2 | **Motivation-Personal belief** | HIV testing enables timely awareness of one’s infection status, allowing protective measures to be taken early to prevent transmission to others. | Excluded | Standardized factor loading below 0.50 |  |  |  |
|  | A3 | **Motivation-Threat perception** | I believe the HIV epidemic in my community is relatively severe. | Retained | — | 0.77 | 0.845 | 0.646 |
|  | A4 | **Motivation-Threat perception** | Delaying testing may lead to a missed opportunity for timely diagnosis and treatment of HIV. | Retained | — | 0.76 |  |  |
|  | A5 | **Motivation-Threat perception** | Delaying testing may result in unknowingly transmitting HIV to others. | Retained | — | 0.88 |  |  |
|  | A6 | **Motivation-Threat perception** | Unprotected anal intercourse, multiple sexual partners, HIV-positive sexual partners, commercial sexual activity, sexually transmitted infections, and substance abuse are all risk factors for HIV infection. | Excluded | Standardized factor loading below 0.50 |  |  |  |
|  | A7 | **Motivation-Personal belief** | HIV testing can provide a sense of certainty regarding one’s own health status. | Retained | — | 0.76 | 0.904 | 0.760 |
|  | A8 | **Motivation-Personal belief** | Undergoing HIV testing is a responsible behavior toward oneself. | Retained | — | 0.96 |  |  |
|  | A9 | **Motivation-Personal belief** | Undergoing HIV testing is a responsible behavior toward others. | Retained | — | 0.89 |  |  |
|  | A10 | **Motivation-Personal belief** | HIV testing can reduce my anxiety about being infected with HIV. | Excluded | Standardized factor loading below 0.50 |  |  |  |
| **Competence** | C1 | **Behavioral skills—Self-efficacy** | Information on HIV testing is easily accessible. | Retained | — | 0.78 | 0.925 | 0.805 |
|  | C2 | **Behavioral skills—Self-efficacy** | I am aware of the proper procedural steps for HIV self-testing. | Retained | — | 0.95 |  |  |
|  | C3 | **Behavioral skills—Self-efficacy** | I understand the significance of the HIV “window period” when interpreting test results. | Retained | — | 0.95 |  |  |
|  | C4 | **Behavioral skills—Self-efficacy** | I can interpret HIV test results and know that if the test is positive, follow-up confirmatory testing should be pursued. | Excluded | Standardized factor loading below 0.50 |  |  |  |
|  | C5 | **Behavioral skills—Self-efficacy** | I am confident in my ability to find avenues to get tested for HIV. | Excluded | Excluded to improve composite reliability |  |  |  |
|  | C6 | **Behavioral skills—Self-efficacy** | I am able to independently decide whether or not to undergo HIV testing. | Excluded | Standardized factor loading below 0.50 |  |  |  |
|  | C7 | **Behavioral skills—Self-efficacy** | I would still undergo HIV testing even if there is a possibility of experiencing discrimination. | Excluded | Standardized factor loading below 0.50 |  |  |  |
|  | C8 | **Behavioral skills—Self-efficacy** | If I encounter problems during HIV testing, I am confident in my ability to seek professional medical advice. | Excluded | Excluded to improve composite reliability |  |  |  |
|  | C9 | **Social motivation** | In my community, the vast majority of people are afraid to get tested for HIV. | Excluded | Insufficient conceptual correspondence with the specific subjective norms |  |  |  |
|  | C10 | **Social motivation** | When friends talk about the importance of HIV testing, it prompts me to take the matter more seriously. | Excluded | Standardized factor loading below 0.50 |  |  |  |
| **Relatedness** | R1 | **Behavioral skills—testing-related barriers** | I have had, or heard of, discriminatory experiences when seeking testing at healthcare facilities, which makes me reluctant to seek testing. | Excluded | Primarily reflected anticipated stigma rather than practical testing-related difficulties |  |  |  |
|  | R2 | **Behavioral skills—testing-related barriers** | Long waiting times at healthcare institutions or clinics discourage me from seeking testing. | Excluded | Standardized factor loading below 0.50 |  |  |  |
|  | R3 | **Behavioral skills—testing-related barriers** | I worry about personal privacy being disclosed or being seen by acquaintances, so I am reluctant to get tested. | Excluded | Primarily reflected anticipated stigma rather than practical testing-related difficulties |  |  |  |
|  | R4 | **Behavioral skills—testing-related barriers** | The high cost of HIV self-testing kits prevents me from undergoing testing. | Retained | — | 0.86 | 0.795 | 0.573 |
|  | R5 | **Behavioral skills—testing-related barriers** | The complicated usage of HIV self-test kits deters me from testing. | Retained | — | 0.83 |  |  |
|  | R6 | **Behavioral skills—testing-related barriers** | I am unsure which channels are available for HIV testing. | Retained | — | 0.54 |  |  |
|  | R7 | **Behavioral skills—testing-related barriers** | I am not aware of accessible nearby testing sites, so I do not get tested. | Excluded | Standardized factor loading below 0.50 |  |  |  |
|  | R8 | **Social motivation-subjective norms** | My community provides sufficient services to support people whose HIV test results are positive. | Retained | — | 0.94 | 0.908 | 0.832 |
|  | R9 | **Social motivation-subjective norms** | If the test is positive, it is easy for me to get information about peer-support groups for follow-up referral. | Retained | — | 0.89 |  |  |
|  | R10 | **Social motivation** | I am concerned that I may be blamed or rejected by my spouse, family, or friends, so I am reluctant to get tested. | Excluded | Insufficient conceptual correspondence with the specific subjective norms |  |  |  |

***All items are answered on a 5-point Likert scale:
1 = Completely disagree, 2 = Some disagree, 3 = Neutral, 4 = Some agree, 5 = Completely agree***

***Items measuring behavioral skills were reverse-scored, such that lower scores indicated higher levels of perceived behavioral skills or capability***
