# Supplementary material for: Identifying factors influencing HIV testing behavior among high-risk elderly men: a mixed-methods study incorporating structural equation modeling and thematic framework
Source: BMC Infect Dis. 2026 Aug 28;26:1671. doi: 10.1186/s12879-026-14305-3 (PMC13587304; doi:10.1186/s12879-026-14305-3)
Supplement: Supplementary file 2 [file 12879_2026_14305_MOESM2_ESM.docx]

## Supplementary **Table 2** Sexual Activity and Sexual Health Characteristics of Participants (n=300)

| **Variables** | **Frequency** | **Proportion（%）** |
| --- | --- | --- |
| **Self-Perceived usual sexual desire*** |  |  |
| Normal sexual desire | 246 | 82 |
| Hypoactive sexual desire | 42 | 14 |
| Hyperactive sexual desire | 6 | 2 |
| Unable to determine | 6 | 2 |
| **Frequency of sexual activity** |  |  |
| 2-3 times per month | 171 | 56.9 |
| Less than 1 time per month | 67 | 22.3 |
| 1 time per week | 36 | 12 |
| 2 times or more per week | 14 | 4.6 |
| Rarely or never | 12 | 4 |
| **Erectile function during sexual activity** |  |  |
| Consistently achieves and maintains erection for intercourse | 252 | 84 |
| Achieves erection but often loses it prematurely | 39 | 13 |
| Has difficulty achieving or maintaining a firm erection | 8 | 2.6 |
| Rarely or never achieves an erection | 1 | 0.3 |
| **Duration of intercourse** |  |  |
| 6-10 minutes | 126 | 42 |
| 3-5 minutes | 113 | 37.6 |
| More than 10 minutes | 50 | 16.6 |
| Less than 2 minutes | 11 | 3.6 |
| **Ever used of sexual enhancement medications** |  |  |
| Never | 239 | 79.6 |
| Seldom | 59 | 19.6 |
| Often | 2 | 0.6 |
| **Have any andrological diseases** |  |  |
| No | 227 | 75.6 |
| Prostate disease | 44 | 14.6 |
| ED/Premature ejaculation/Sexually transmitted diseases | 29 | 9.6 |
| **Degree of sexual satisfaction** |  |  |
| Basically satisfied | 235 | 78.3 |
| Basically not satisfied | 58 | 19.3 |
| Seriously not satisfied | 7 | 2.3 |
| **Main reasons for unmet sexual needs^#^** |  |  |
| Lack of sexual interest from spouse | 20 | 30.8 |
| Lack of sexual partner | 27 | 41.5 |
| One's own insufficient sexual ability | 13 | 20.0 |
| Others | 5 | 7.7 |

********Self-perceived usual sexual desire was assessed based on participants’ own evaluation of their usual level of sexual desire. This item was used as a descriptive self-reported measure rather than a clinical diagnosis.*

***^#^*** *Reasons for unmet sexual needs were assessed only among participants who reported unmet sexual needs (n = 65).*
