# Supplementary material for: Identifying factors influencing HIV testing behavior among high-risk elderly men: a mixed-methods study incorporating structural equation modeling and thematic framework
Source: BMC Infect Dis. 2026 Aug 28;26:1671. doi: 10.1186/s12879-026-14305-3 (PMC13587304; doi:10.1186/s12879-026-14305-3)
Supplement: Supplementary file 3 [file 12879_2026_14305_MOESM3_ESM.docx]

## Supplementary **Table 3** Covariate-adjusted sensitivity analysis of the structural equation model

| ****Outcome variable**** | ****Predictor**** | ****Direct effect**** | *****P*-value**** | ****95% CI**** | ****Indirect effect**** | *****P*-value**** | ****95% CI**** | ****Total effect**** | *****P*-value**** | ****95% CI**** |
| --- | --- | --- | --- | --- | --- | --- | --- | --- | --- | --- |
| **Self-efficacy** | **Belief** | 0.212 | 0.001 | **[0.094, 0.332]** | — | — |  | 0.212 | 0.001 | [0.094, 0.332] |
|  | **Threat perception** | 0.192 | 0.009 | **[0.050, 0.326]** | — | — |  | 0.192 | 0.009 | [0.050, 0.326] |
|  | **Information** | 0.142 | 0.001 | **[0.061, 0.233]** | — | — |  | 0.142 | 0.001 | [0.061, 0.233] |
| **Behavioral skills** | **Threat perception** | 0.710 | < 0.001 | [0.602, 0.807] | — | — |  | 0.710 | <0.001 | [0.602, 0.807] |
|  | **Information** | −0.131 | 0.001 | [−0.224, −0.048] | — | — |  | −0.131 | 0.001 | [-0.224, -0.048] |
| **Ever-tested status** | **Belief** | — | — |  | 0.036 | 0.001 | [0.010,0.072] | 0.036 | 0.001 | [0.012, 0.072] |
|  | **Threat perception** | 0.455 | 0.001 | [0.191, 0.738] | −0.116 | 0.136 | [-0.313,0.028] | 0.339 | 0.001 | [0.142, 0.526] |
|  | **Subjective norms** | 0.275 | 0.004 | [0.094, 0.464] | — | — |  | 0.275 | 0.004 | [0.094, 0.464] |
|  | **Information** | −0.259 | 0.001 | [−0.372, −0.123] | 0.052 | 0.001 | [0.018,0.099] | −0.207 | 0.002 | [-0.313, -0.079] |
|  | **Self-efficacy** | 0.172 | 0.002 | [0.062, 0.286] | — | — |  | 0.172 | 0.002 | [0.062, 0.286] |
|  | **Behavioral skills** | −0.210 | 0.017 | [−0.466, −0.039] | — | — |  | −0.210 | 0.017 | [-0.466, -0.039] |
|  | **Age** | 0.094 | 0.048 | [0.001, 0.202] | — | — |  | 0.094 | 0.048 | [0.001, 0.202] |
|  | **Residence** | −0.040 | 0.520 | [−0.182,  0.093] | — | — |  | −0.040 | 0.520 | [-0.182, 0.093] |
|  | **Junior high school** | −0.016 | 0.762 | [-0.114, 0.086] | — | — |  | −0.016 | 0.762 | [-0.114, 0.086] |
|  | **Senior high school or above** | −0.042 | 0.377 | [-0.133, 0.051] | — | — |  | −0.042 | 0.377 | [-0.133, 0.051] |
|  | **Occupation** | 0.113 | 0.097 | [-0.020, 0.251] | — | — |  | 0.113 | 0.097 | [-0.020, 0.251] |
|  | **Monthly income(CNY) 1,001–3,000** | 0.007 | 0.863 | [-0.120, 0.144] | — | — |  | 0.007 | 0.863 | [-0.120, 0.144] |
|  | **Monthly income(CNY)**  **> 3,000** | 0.031 | 0.592 | [-0.099, 0.191] | — | — |  | 0.031 | 0.592 | [-0.099, 0.191] |
|  | **Local residence** | 0.123 | 0.025 | [0.014, 0.243] | — | — |  | 0.123 | 0.025 | [0.014, 0.243] |

***Note: Effects are standardised coefficients. P values were estimated using two-tailed bias-corrected Bootstrap tests based on 5,000 resamples.***

***Model fit: χ²/df = 2.508; CFI = 0.918; TLI = 0.891; RMSEA = 0.071 (90% CI: 0.063–0.079).***

***“—” indicates that no corresponding path was specified.***

***Items measuring behavioral skills were reverse-scored, such that lower scores indicated higher levels of perceived behavioral skills or capability.***
